# Detoxification of Aflatoxin-Contaminated Maize by Neutral Electrolyzed Oxidizing Water

**DOI:** 10.3390/toxins7104294

**Published:** 2015-10-23

**Authors:** Samantha Jardon-Xicotencatl, Roberto Díaz-Torres, Alicia Marroquín-Cardona, Tania Villarreal-Barajas, Abraham Méndez-Albores

**Affiliations:** 1 UNAM–FESC. Campus 4. Multidisciplinary Research Unit L14 (Food, Mycotoxins and Mycotoxicosis), Cuautitlan Izcalli 54714, Mexico; E-Mail: mvz.jardon@gmail.com; 2 UNAM–FESC. Campus 4. Multidisciplinary Research Unit L9 (Toxicology and Genetics), Cuautitlan Izcalli 54714, Mexico; E-Mail: diaztorres_r@hotmail.com; 3 UANL–FMVZ. Agropecuary Sciences Campus, General Escobedo 66050, Mexico; E-Mail: aliciamarroquin@hotmail.com; 4 Esteripharma SA de CV. Atlacomulco 50450, Mexico; E-Mail: tvillarreal@esteripharma.com.mx

**Keywords:** maize, aflatoxins, neutral electrolyzed oxidizing water, detoxification

## Abstract

Aflatoxins, a group of extremely toxic mycotoxins produced by *Aspergillus flavus*, *A. parasiticus* and *A. nomius*, can occur as natural contaminants of certain agricultural commodities, particularly maize. These toxins have been shown to be hepatotoxic, carcinogenic, mutagenic and cause severe human and animal diseases. The effectiveness of neutral electrolyzed oxidizing water (NEW) on aflatoxin detoxification was investigated in HepG2 cells using several validation methodologies such as the 3-(4,5-dimethylthiazol-2-yl)-2,5-diphenyltetrazolium bromide assay, the induction of lipid peroxidation, the oxidative damage by means of glutathione modulation, the Ames test and the alkaline Comet assay. Our results showed that, after the aflatoxin-contaminated maize containing 360 ng/g was soaked in NEW (60 mg/L available chlorine, pH 7.01) during 15 min at room temperature, the aflatoxin content did not decrease as confirmed by the immunoaffinity column and ultra performance liquid chromatography methods. Aflatoxin fluorescence strength of detoxified samples was similar to untreated samples. However, aflatoxin-associated cytotoxicity and genotoxicity effects were markedly reduced upon treatment. According to these results, NEW can be effectively used to detoxify aflatoxin-contaminated maize.

## 1. Introduction

Aflatoxins, a group of acutely toxic metabolites produced mainly by strains of *Aspergillus flavus* Link, *Aspergillus parasiticus* Speare and *Aspergillus nomius* Kurtzman *et al.* [1,2], have close chemical molecular structures (difuranocoumarins) and form a unique group of naturally occurring compounds. Aflatoxin B_1_ (AFB_1_)—the most commonly encountered toxic metabolite in this group—causes severe liver damage and has higher toxicity and carcinogenicity than other aflatoxins; as a result, it has been classified as a human liver carcinogen (Group 1) by the International Agency for Research on Cancer [3].

Primary liver cancer, which consists predominantly of hepatocellular carcinoma (HCC) is the fifth most common cancer and the third most frequent cause of cancer mortality worldwide [4]. Epidemiological studies conducted in areas with high HCC incidence mainly investigated the possible association between dietary exposure to AFB_1_ and the occurrence of HCC [5]. Once ingested by humans, AFB_1_ is metabolized to an active intermediate named AFB_1_*-exo*-8,9-epoxide, which can bind to DNA and induce damage, producing the characteristic mutation at codon 249 of the tumor-suppressor p53 gene [6]. This particular mutation has been observed for up to 60% of HCC tumors in aflatoxin-endemic areas [7].

The increasing number of reports on the presence of aflatoxins in food and feedstuffs dictates the need for decontamination procedures; such procedures should not only reduce the mycotoxin content to “*safe*” levels—below regulatory limits—but should also have the following characteristics: easy to use, inexpensive and free of the potential for forming compounds that are still toxic or compromising the nutritional value of the treated commodity [8]. A number of methods have been investigated either to inactivate, remove or destroy the toxin in order to reduce or eliminate the toxic effects. These can be classified into physical, chemical or biological methods [9]. Aflatoxins can be transformed into less mutagenic/toxic substances when treated with different chemical compounds. However, most of the chemical processes that have been investigated are impractical (required to be carried out under extreme conditions of temperature and pressure), unsafe (due to the formation of toxic residues) and compromise the nutritional, sensory and functional properties of the product. For these reasons, research into secure, effective, practical, inexpensive and environmentally friendly methods for aflatoxin detoxification is a high priority for the food industry.

Electrolyzed oxidizing water (EOW) is produced by electrolysis of pure water—with no added chemicals, except for sodium chloride—in an electrolysis equipment, where anodes and cathodes are separated by a non-selective membrane. In the anode, chloride ions and water molecules are transformed into chlorine oxidants such as hypochlorous acid (HOCl), hypochlorite ions (ClO^−^) and chlorine (Cl_2_) [10]. In the process, two types of water are generated: neutral electrolyzed water (NEW) and acidic electrolyzed water (AEW). The antimicrobial mechanism of both products depend mainly on three physicochemical properties: pH, oxidation-reduction potential (ORP) and available chlorine concentration (ACC) [11]. AEW has considerable biocidal activity, but rapidly loses ORP and Cl_2_—through the evolution of chlorine gas—thus reducing its effectiveness. On the contrary, the loss of chlorine oxidants and ORP from near-neutral electrolyzed water (which contains primarily HOCl) is considerably lower than acidic solutions. Consequently, NEW could be used for the development of safer and more socially acceptable methods for aflatoxin detoxification, since NEW minimize human health and safety concerns, reduces corrosion and limits toxic side effects. Increasing evidence has suggested that EOW (particularly NEW, pH 5.6, ORP 836.4 mV and ACC 83.7 mg/L) has strong antifungal activity against *A. flavus* and is also effective to decontaminate AFB_1_ in peanuts [12]. However, the application of NEW (pH 7.01, ORP 766 mV and ACC 60 mg/L) to detoxify aflatoxin-contaminated maize as well as the toxicological evaluation of the chemically treated commodity have not been reported yet. Consequently, the present research was conducted to determine the safety and efficacy of the detoxification procedure based on the use of NEW to minimize aflatoxin-associated cytotoxicity and genotoxicity effects on *in vitro* assays. For this purpose, the inhibition of cell viability, the formation of malondialdehyde as a marker of aflatoxin-induced lipid peroxidation and the oxidative damage using glutathione modulation were conducted to evaluate the cytotoxic activity of aflatoxins using hepatocellular carcinoma epithelial cells (HepG2). Furthermore, the *Salmonella*-microsomal screening system (Ames test) by the use of the *S. typhimurium* tester strain TA-100 and the single cell gel electrophoresis (alkaline Comet assay) with human lymphocytes were conducted to evaluate the genotoxic potential of the aflatoxins.

## 2. Results and Discussion

### 2.1. Efficacy of NEW to Detoxify Aflatoxin-Contaminated Maize

Three physicochemical properties of NEW and distilled water (DW) are listed in Table 1. After electrolysis and up to six months later, the pH value (pH 7.01), ORP (766 mV) and ACC (60 mg/L) of NEW were completely stable. By contrast, DW was slightly acidic (pH 5.60), had a low ORP (459 mV) and an extremely low ACC (<1 mg/L). The most significant differences in the physicochemical properties were in ORP and ACC; in the case of NEW, available chlorine acts as a powerful oxidant, which was totally dependent on the electrolysis conditions.

**Table 1:** Some physicochemical properties of neutral electrolyzed water (NEW) and distilled water (DW).

Parameter	NEW	DW
PH	7.01 ± 0.04	5.60 ± 0.15
ORP (mV)	766 ± 3.4	459 ± 2.5
ACC (mg/L)	60 ± 0.47	<1

Mean of three replicates ± standard error, Abbrev: ORP, oxidation-reduction potential; ACC, available chlorine concentration; NEW, neutral electrolyzed oxidizing water; DW, distilled water.

**Figure 1:**
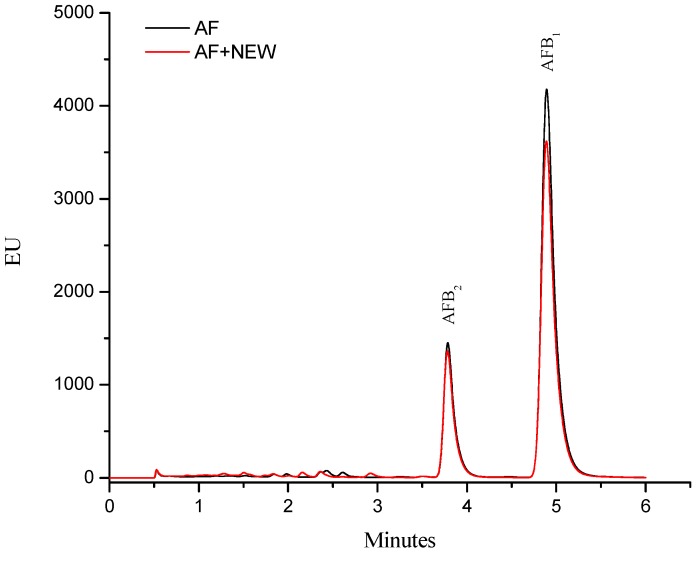
The fluorescence of aflatoxins from contaminated maize samples: control (AF) and treated with neutral electrolyzed oxidizing water (AF + NEW).

The aflatoxin quantification indicated that inoculated maize contained 360 ± 10 ng/g. This total aflatoxin value represents content that may be found in commercial maize used to produce food in several regions of Mexico [13]. Moreover, the *A. flavus* strain used in this research produces both AFB_2_ and AFB_1_, and the retention time (Rt) values were 3.78 and 4.88 min, respectively. AFB_1_ was the most abundant toxin produced by this strain, accounting for up to 80% (≈290 ng/g) of the total aflatoxin content. After treatment with NEW, the aflatoxin content did not significantly decrease (determined with the immunoaffinity column and ultra performance liquid chromatography methods via fluorescence measurement); in consequence, aflatoxin fluorescence strength of NEW–detoxified samples was almost similar to the control samples (Figure 1).

These results suggest that this particular detoxification procedure does not reduce the aflatoxin content (measured as loss of fluorescence). Xiong *et al.* [14] reported that AlEW (pH 11.6, ORP −872 mV) has no effect on fluorescence of AFB_1_. However, NEW (pH 5.6, ORP 836.4 mV and ACC 83.7 mg/L) or AEW (pH 2.5, ORP 1117.3 mV and ACC 80.2 mg/L) significantly diminished the peak of fluorescence of the trifluoroacetylated AFB_1_. Our results are partially in agreement with those reported by Xiong *et al.* [14], since it is well known that the first step in the degradation of aflatoxins during alkaline or acidic treatments is likely to be the base-induced lactone ring-opening of the aflatoxin molecule, yielding a water soluble salt including decarboxilation. Thus, aflatoxin fluorescence—attributable to the coumarin moiety—disappears in alkaline or acidic conditions [15,16,17].

Xiong *et al.* [14] demonstrated that HOCl—the main available chlorine form in near-neutral electrolyzed oxidizing water—underwent addition to AFB_1_. The authors indicated that the conversion product was positively charged with chemical formulae C_17_H_13_ClO_7_. Otherwise, the −Cl and −OH groups were added to the C_8_ and C_9_ atoms in the terminal furan ring of the aflatoxin molecule. We are in close agreement with this conclusive evidence, since it is well known that hypochlorous acid can react with certain compounds containing double bonds producing chlorohydrins. Carr *et al.* [18] reported that the reaction of HOCl (a strong oxidant generated by the myeloperoxidase system of neutrophils and monocytes) with the double bonds of unsaturated membrane lipids produce alpha or beta chlorohydrin isomers. Our research group also reported that aqueous citric acid can also lead to hydration of the AFB_1_molecule at the 8,9-olefinic bond of the terminal furan ring to form AFB_2a_ (hydroxydihydro-aflatoxin B_1_) which has less than 1/200 toxicity than AFB_1_ [19]. Comparison of the mutagenic activity of aflatoxins with the double bond in the terminal furan ring seems to support the involvement of this functional group in toxicological action. Thus, the hepatocarcinogenic potential of AFB_1_ is greatly reduced when the 8,9-double bond is hydrogenated; however, the double bond does not seem to be the sole molecular site which determines mutagenic activity. Alterations occurring elsewhere in the molecule invariably result in the reduction of toxicity. It has been reported that alterations in the lactone ring (hydrolyzed during acidic conditions) result in a significant lowering of mutagenic potential, despite the presence of an intact double bond [17].

Due to the fact that AFB_1_ is mainly produced by *A. flavus* strains, we suggest that NEW reacts with the double bond in the terminal furan ring of the AFB1 molecule to yield 8-chloro-9-hydroxy-aflatoxin B_1_, which is in agreement with Xiong *et al.* [14]. The most important factor in AFB_1_ transformation is the high level of ACC, taking into account that NEW contains primarily hypochlorous acid (≈95%), the hypochlorite ion (≈5%) and trace amounts of Cl_2_ [20]. In this research, NEW contained an initial ACC of 60 ± 0.47 mg/L; however, the post–reaction ACC was 34.5 ± 1.1 mg/L. This reduction represents about 43% of the ACC.

In general, it is expected that electrolyzed oxidizing solutions (such as AEW, AlEW or NEW) would eliminate the aflatoxin-associated cytotoxicity and genotoxicity effects due to reaction with the double bond in the terminal furan ring. However, AEW and AlEW have limited potential for long-term applications in the food industry due to its strong acidity or alkalinity. Buck *et al.* [21] stated that AEW is slightly phytotoxic to some species of bedding plants and may also cause corrosion. On the contrary, NEW is non-toxic, non-corrosive and safe due to its reverting capacity into ordinary water when diluted with tap water or reverse osmosis. Thus, the application of NEW to detoxify aflatoxin-contaminated maize offers many advantages over other chemical methods, including less adverse chemical residues, secure, energy-saving, cost-effective and environmentally-friendly.

### 2.2. Cytotoxic Effects

**Figure 2:**
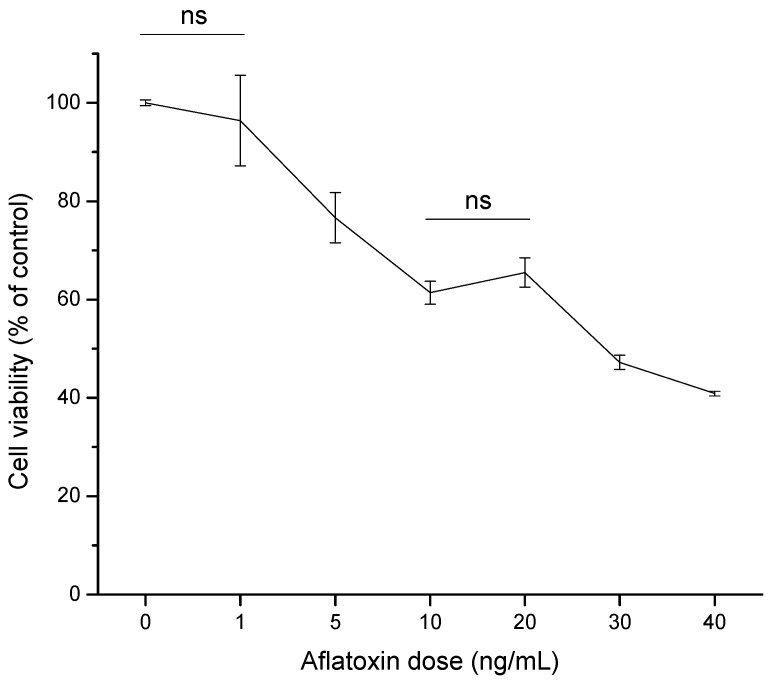
Dose–response curve of aflatoxins in hepatocellular carcinoma epithelial cells (HepG2) after 4 h of exposure. Cell viability is expressed as a percentage of control. Data were expressed as mean values ± standard error of three independent experiments (ns = not significant).

The human HCC cell line (HepG2) has been widely used as a model system to evaluate toxic effects of various hepatocarcinogenic substances. It is well known that cultured cells show decreased cell survival in the presence of AFB_1_, which could be related to either DNA damage caused by the toxin or direct impairment of specific protein functions [22,23]. In this research, the cytotoxic effect of aflatoxins (0 to 40 ng/mL) in HepG2 cell line as measured by the MTT [3-(4,5-dimethylthiazol-2-yl)-2,5-diphenyltetrazolium bromide] assay was assessed. After 4 h of exposure, aflatoxins induced a notable decrease of viable cells in a dose-dependent manner, indicating that the HepG2 cell line was sensitive to the toxic effects of the tested mycotoxin (Figure 2). At 10 and 20 ng/mL aflatoxins, both doses demonstrated no significant differences in HepG2 cell viability, presenting viability percentages up to 65%. However, a significant effect was observed in cell viability within 4 h of exposure at 30 ng/mL aflatoxins, reaching the 50% inhibitory concentration (IC_50_). Furthermore, at the highest aflatoxin dose tested (40 ng/mL), HepG2 viability decreased up to 40% of the control level. Based on the determined viability percentages of the dose-response study, the dose of 6 ng/mL (80% estimated viability using a polynomial function, *R*^2^ = 0.93) was selected to evaluate the cytotoxic effects of the aflatoxin–contaminated maize samples (untreated and treated with NEW).

#### 2.2.1. Cell Viability (MTT Assay)

**Figure 3:**
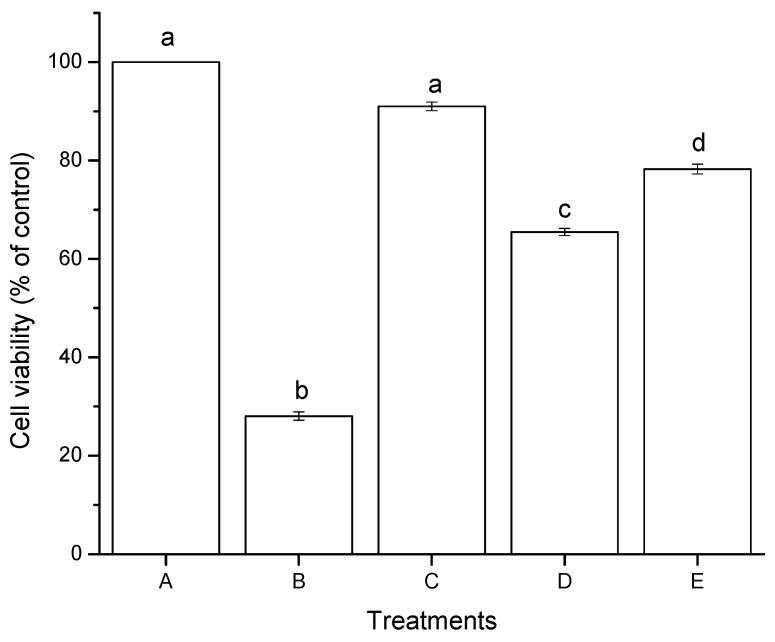
Cytotoxic effects of aflatoxins in hepatocellular carcinoma epithelial cells (HepG2) after 4 h of exposure. (**A**) negative control (DMSO); (**B**) positive control (H_2_O_2_, 30 mM); (**C**) NEW (17 µL/mL); (**D**) untreated aflatoxins (AF at 6 ng/mL); (**E**) aflatoxins treated with NEW (AF + NEW at 6 ng/mL). Mean values ± standard error of three independent experiments. Bars with the same letter are not significantly different (Tukey’s test, *p* > 0.05).

The effect of aflatoxins in HepG2 cells after 4 h of incubation as measured by the MTT assay is shown in Figure 3. NEW alone showed no significant difference in cell viability, with a percentage closest to the negative control (91%). However, significant differences were observed between aflatoxins (untreated and treated with NEW at 6 ng/mL). Untreated aflatoxins (AF) caused a notable decrease in cell viability, reaching values of approximately 65%. However, higher viability percentages were observed for aflatoxins treated with NEW (AF + NEW), showing viable cells in the upper 78% of the control level. Positive control using hydrogen peroxide (30 mM) had the lowest cell viability percentage (28%). In general, the MTT assay reveals that both aflatoxin samples showed considerable reductions in cell viability, up to 35% for AF and up to 22% for AF + NEW (Figure 3).

The cytotoxicity of aflatoxins has been investigated in some *in vitro* systems especially in hepatic cells (HepG2), where different IC_50_ values were reported at different exposure times. Kuilman *et al.* [24] proved the cytotoxicity of AFB_1_ in bovine hepatocytes. No cytotoxicity was found after 2 and 8 h of incubation at concentrations ranging from 1 to 16 μM AFB_1_. However, after 24 h of incubation, a clear concentration-dependent increase in cytotoxicity was observed, resulting in less than 40% viable cells at 16 μM AFB_1_. McKean *et al.* [25] evaluated the cytotoxic effects of AFB_1_ at doses of 0.01 to 100 µM in the human hepatoma cell line (HepG2) as measured by the tetrazolium dye-based WST–1 assay. At 24 h of exposure, the IC_50_ value was estimated at 1 µM. Liu *et al.* [26] reported that after 24 h of exposure of HepG2 cells to 100 µM AFB_1_, the cell viability was reduced more than 50%. Xiong *et al.* [14] determined the cytotoxicity of AFB_1_ in HepG2 cells using the MTT assay. After 48 h of exposure, the determined IC_50_ value was less than 0.015 µM (4.68 ng/mL). The cytotoxic effects of the 8-chloro-9-hydroxy-aflatoxin B_1_ compound—the conversion product of electrolyzed oxidizing water (EOW) treatment—was also evaluated. This compound was found to be less cytotoxic than AFB_1_ after 48 h of exposure, even at the maximum concentration tested (1.20 µM). The examined IC_50_ value of the 8-chloro-9-hydroxy-aflatoxin B_1_ was estimated at 150 mM. Therefore, the authors suggested that this compound may be considered nontoxic. These results are consistent with our research, since aflatoxins (untreated and treated with NEW at 6 ng/mL) inhibit cell viability at different rates. The elevated viability percentage of AF + NEW is associated with reduced cytotoxicity as measured by the MTT assay. Consequently, the treatment of aflatoxin-contaminated maize with NEW results in reduced cytotoxicity, even though the content of aflatoxin on the detoxified grain was not significantly reduced.

#### 2.2.2. Lipid Peroxidation (Oxidative Stress)

**Figure 4:**
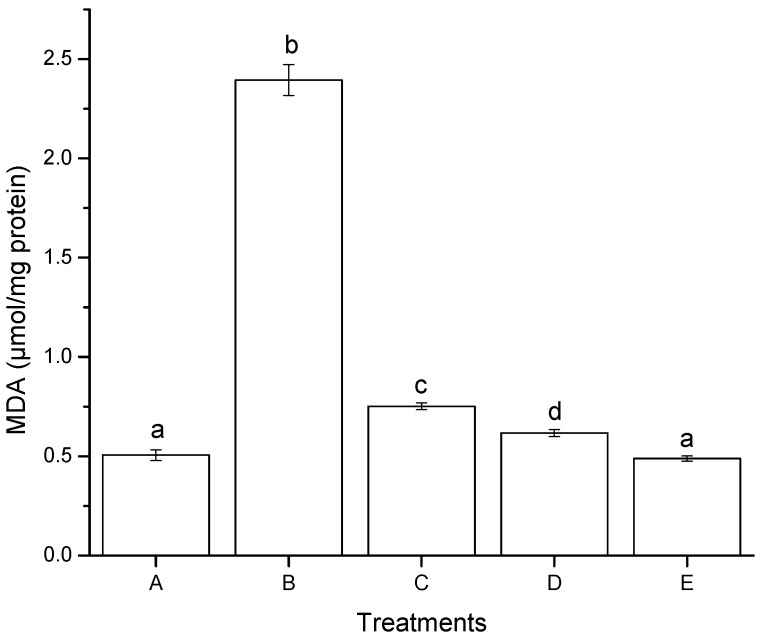
Lipid peroxidation induced after 4 h of exposure to aflatoxins in hepatocellular carcinoma epithelial cells (HepG2), measured by production of malondialdehyde (MDA). (**A**) negative control (DMSO); (**B**) positive control (H_2_O_2_, 30 mM); (**C**) NEW (17 µL/mL); (**D**) untreated aflatoxins (AF at 6 ng/mL); (**E**), aflatoxins treated with NEW (AF + NEW at 6 ng/mL). MDA results were normalized according to total protein concentration. Mean values ± standard error of three independent experiments. Bars with the same letter are not significantly different (Tukey’s test, *p* > 0.05).

Oxidative stress has been defined as a disturbance in the prooxidant-antioxidant balance, resulting in potential cell damage. The reactive oxygen species (ROS) and free radicals—initiated by lipid peroxidation—also induce a wide range of lesions on target cells. Numerous methods are described for cytotoxicity studies, especially for mycotoxins [27], including the inhibition of cellular growth, the capacity of cells to synthesize cellular macromolecules, and the ability of the toxic agent to induce lipid peroxidation—one of the cellular pathways involved in oxidative damage. In this research, after 4 h of incubation with AF + NEW, the malondialdehyde (MDA) detected in HepG2 cells decreased significantly in comparison to AF (Figure 4).

The MDA concentration for AF + NEW was 0.49 µmol/mg protein, similar to the negative control (0.51 µmol/mg total protein), while AF had 0.62 µmol/mg protein MDA concentration. Furthermore, the MDA concentration increased significantly when HepG2 cells were exposed to NEW alone (up to 0.75 µmol/mg protein). These findings suggest that NEW (containing primarily HOCl) modulate cellular signals in hepatic cells that promote the production of ROS causing a significant increment in lipid peroxidation. These results are in accordance with Güngör [28] who investigated the effect of HOCl on MDA formation in lung epithelial A549 cells. Higher MDA levels in cells treated with HOCl (50 μM) as measured by thiobarbituric acid reactive species (TBARS) formation were reported. This increase in MDA formation can be assigned to HOCl-induced lipid peroxidation.

A number of authors have reported increments in lipid peroxidation due to mycotoxin exposure. Vázquez-Durán *et al.* [29] reported that aflatoxins from contaminated maize (141.5 ng/g) increased the MDA concentration from the basal value of 0.05 μmol/mg protein in the negative control to 6.05 μmol/mg protein, using monkey kidney cells (Vero cells). Abado-Becognee *et al.* [30] reported that fumonisin B_1_ (FB_1_) was found to be a potent inducer of MDA in Vero cells. At 0.14 µM, FB_1_ induced 0.496 nmoles of MDA/mg protein compared to the control level (0.134 nmoles of MDA/mg protein). Abid-Essefi *et al.* [31] also monitored MDA formation in Vero and Caco-2 cells exposed to zearalenone (ZEN). These authors reported that after 24 h of incubation with ZEN (40 µM), the MDA detected in Vero cells increased from 270 nmol/mg protein to 484 nmol/mg protein. In the same conditions, similar results were found in Caco-2 cells. The MDA production increased from basal value of 323 nmol/mg protein in controls to 515 nmol/mg protein. Lee *et al.* [32] investigated AFB_1_-induced oxidative stress in HepG2 cells using a volatile extract from Allii Fistulosi Bulbus (VEAF). Treatment with 10 μM AFB_1_ had influence on the cellular TBARS levels, which increased to 25.8% with AFB_1_. However, the pretreatment with 1 and 10 μg/mL of VEAF, significantly reduced the TBARS formation by 48.0% and 59.7%, respectively. It is well known that mycotoxins induce lipid peroxidation in a concentration-dependent manner. Lipid peroxidation alters the structure and function of the cellular membrane and blocks cellular metabolism leading to cytotoxicity. Lipid peroxidation may also be related to the disturbance of cell signalling processes, genotoxicity, mutagenicity and tumor promotion, since it has been shown that the diene conjugates—produced during lipid peroxidation—can interact with DNA. As a result, several adducts with nucleobases such as: 1,*N*^6^-ethenoadenine and 3,*N*^4^-ethenocytosine are formed [33]. Our findings showed that AF induced greater oxidative damage by enhancing lipid peroxidation in HepG2 cells; however, AF + NEW did not induce significant MDA formation at the same concentration tested (6 ng/mL). These results emphasize the notable oxidative damage caused by the non-treated aflatoxin molecules as compared to those treated with NEW. Therefore, it could be expected that aflatoxin molecules detoxified with NEW would not stimulate the carcinogenic process, as suggested by El Ghissassi *et al.* [33].

#### 2.2.3. Reduced Glutathione (GSH) Modulation

**Figure 5:**
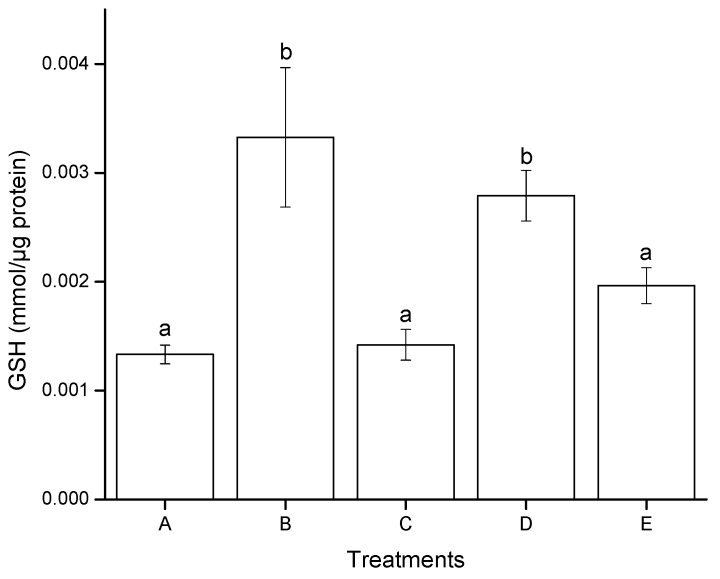
Modulation of GSH in hepatocellular carcinoma epithelial cells (HepG2), induced after 4 h of exposure to aflatoxins. (**A**) negative control (DMSO); (**B**) positive control (H_2_O_2_, 30 mM); (**C**) NEW (17 µL/mL); (**D**) untreated aflatoxins (AF at 6 ng/mL); (**E**) aflatoxins treated with NEW (AF + NEW at 6 ng/mL). GSH results were normalized according to total protein concentration. Mean values ± standard error of three independent experiments. Bars with the same letter are not significantly different (Tukey’s test, *p* > 0.05).

Figure 5 illustrates the effect of aflatoxins on GSH modulation in HepG2 cells after 4 h of exposure. A significant difference was found only for AF treatment, the GSH production increased from 0.0013 mmol/µg protein in negative controls (DMSO) to 0.0027 mmol/µg protein. On the contrary, no significant differences were observed in GSH level between AF + NEW, NEW and the negative control. In general, the redox status in cells exposed to AF + NEW and NEW alone was not affected; consequently, a normal GSH level was enough to modulate the prooxidant–antioxidant balance. In contrast, positive control (H_2_O_2_, 30 mM) significantly increased the GSH level reaching values up to 0.0033 mmol/µg protein.

Vázquez-Durán *et al.* [29] evaluated the effect of aflatoxin extracts on GSH modulation in Vero cells after 4 h of exposure. The cell line presented a markedly depletion of GSH level in a dose-dependent manner. However, two aflatoxin treatments increased the GSH levels to 103% and 66%, respectively (compared to the negative control). These authors stated that the significant increment in the GSH level could be possibly due to the presence of high oxidative aflatoxin degradation products—resulting from the thermal-alkaline process—which allowed the cells to over-express GSH in order to modulate their intracellular redox status. Our results are consistent with the findings of Vázquez-Durán *et al.* [29]. It is well known that the Phase I and/or Phase II enzymes involved in detoxification are induced when cells are confronted with a high xenobiotic load. This leads to a notable extra-accumulation of enzyme/conjugate (including glucuronidation, sulfation, glutathione and amino acid conjugation), especially in the first few hours after exposure for a faster rate of xenobiotic detoxification. Moreover, evidence supports the idea that the rate of conjugation of GSH with AFB_1_ exo-epoxide is an important factor in determining the species variation in risk to aflatoxins [34].

### 2.3. Genotoxic Effects

#### 2.3.1. Ames Test

The range of revertants per plate for the bacterial strain TA-100 is 100 to 200. The basal number of revertants per plate—with metabolic activation—was 107 ± 16, falling within the standard range. AFB_1_ standard at 2.5, 5, 10 and 20 ng/plate were used as positive controls for mutation induction, and the results agree with our previous results [29]. AFB_1_ at 20 ng/plate was not included in Table 2 because of its high value of induced mutations (903 ± 73). In general, AF + NEW reduced the number of revertants to 103 ± 11, and no significant differences were observed as compared to the negative control (DMSO); whereas for AF, the number of revertants was 216 ± 19, statistically similar to the positive control at 5 ng/plate. In the case of NEW, the number of revertants per plate after treatment of the bacterial strain TA-100 was 111 ± 17 (Table 2).

The Ames test demonstrated that aflatoxins treated with NEW did not exhibit mutagenic activity in the presence of rat liver S9 mix, as compared with untreated aflatoxins. It is well known that AFB_1_ has shown increased mutation frequency—mainly in the tester strain TA-100—after microsomal activation. This indicates that this molecule is a pro-mutagen, which is converted into a potential carcinogen after metabolic activation. Therefore, AFB_1_ is able to induce base pair substitutions in one of the GC pairs [35]. Punvittayagul [36] evaluated the antimutagenic capacity of hydrophilic and lipophilic extracts of Thai northern purple rice against AFB_1_, using the *S. typhimurium* strains TA-98 and TA-100. At a concentration of 0.1 mg/plate, the methanolic extract of rice seed moderately inhibited AFB_1_-induced mutagenesis (69% inhibition). On the other hand, Xiong [14] reported that aflatoxins treated with NEW produced 100 ± 11 revertants/plate when using the tester strain TA-100. Slightly higher values were reported for aflatoxins treated with AEW (162 ± 13 revertants/plate); however, no significant differences were observed as compared to the negative control (135 ± 22 revertants/plate). These authors conclude that aflatoxins treated with EOW have minor potential to induce mutagenicity, which is in accordance with our Ames test results.

**Table 2:** Mutagenic response of the *S. typhimurium* tester strain TA-100 to aflatoxins.

Treatment	Revertants/Plate
A	107 ± 16 ^a^
B	161 ± 25 ^b^
C	223 ± 50 ^c^
D	344 ± 4 ^d^
E	111 ± 17 ^a^
F	216 ± 19 ^c^
G	103 ± 11 ^a^

A—negative control (DMSO); B—positive control (AFB_1_, 2.5 ng/plate); C—positive control (AFB_1_, 5 ng/plate); D—positive control (AFB_1_, 10 ng/plate); E—NEW (10 µL/plate); F—untreated aflatoxins (AF at 5 ng/plate); G—aflatoxins treated with NEW (AF + NEW at 5 ng/plate). Mean values ± standard error of three independent experiments. The same letters indicate no significant difference in mean values of revertants per plate (Tukey’s test, *p* > 0.05).

AFB_1_ is considered to be the most carcinogenic of the aflatoxins family—followed by AFG_1_—and requires oxidation of the 8,9-double bond to yield the biologically active AFB_1_-8,9-epoxide (a highly reactive ultimate carcinogen) which can react with DNA. However, it has been observed that the hepatocarcinogenicity of AFB_1_ is greatly reduced when the 8,9-double bond is hydrogenated to yield AFB_2_. Wong and Hsieh [37] reported that the mutagenicity of AFB_2_ was 500 fold less than that of AFB_1_, due to the lack of the double bond between C_8_ and C_9_. Considering that NEW reacted with the double bond between C_8_ and C_9_ to produce 8-chloro-9-hydroxy-aflatoxin B_1_, the hepatocarcinogenic potential of AFB_1_ greatly diminished, as confirmed with the carcinogenic data of the bacterial detection system used in this research.

#### 2.3.2. Comet Assay

**Figure 6:**
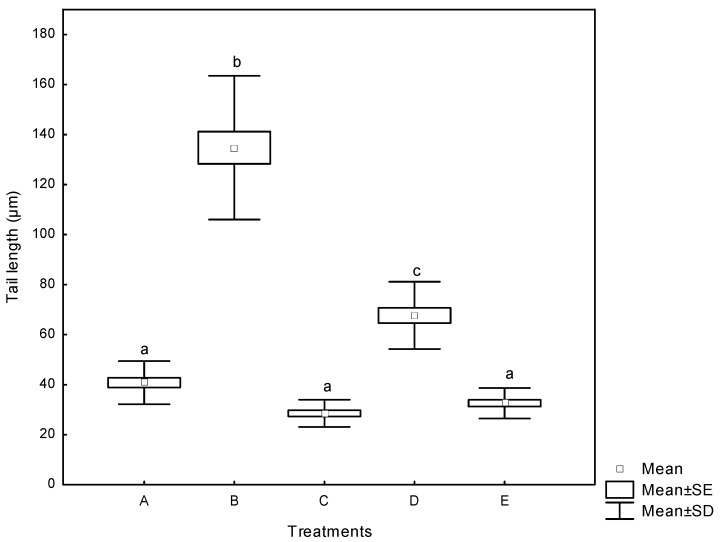
DNA damage in human lymphocytes treated with aflatoxins as measured by the tail length (the migration of DNA in the tail). (**A**) negative control (DMSO); (**B**) positive control (H_2_O_2_, 30 mM); (**C**) NEW (4 µL); (**D**) untreated aflatoxins (AF at 6 ng/mL); (**E**) aflatoxins treated with NEW (AF + NEW at 6 ng/mL). Box and whiskers with the same letter are not significantly different (Tukey’s test, *p* > 0.05). The spontaneous revertants have already been subtracted.

The Comet assay (single cell gel electrophoresis) is an attractive technique increasingly employed in biological systems for evaluating DNA damage. It is a quick, simple, sensitive, reliable and fairly inexpensive method of measuring DNA damage [38]. The alkaline Comet assay, in addition to measuring DNA strand breakage, measures alkali-labile sites or intermediates in base- or nucleotide- excision repair [39]. The DNA damage in human lymphocytes treated with aflatoxins reported as the tail length (the migration of DNA in the tail) is presented in Figure 6. AF significantly increased the tail length from basal value of 41 µm in negative controls (DMSO) to 68 µm. For AF+NEW, the tail length was 33 µm, statistically similar to NEW (33 µm) and DMSO treatments. However, positive control (H_2_O_2_, 30 mM) substantially increased the tail length, reaching values up to 135 µm. Our results suggest a markedly different susceptibility to DNA damage in human lymphocytes from exposure to aflatoxins (untreated and treated with NEW), under *in vitro* conditions. The cell viability was determined by FDA/BrEt, and only cultures with cell viability of more than 80% were used for analysis.

During electrophoresis in the Comet assay and depending on the treatment, fragments of DNA migrate away from the DNA core to form a fast moving tail as seen in Figure S1. Using the Comet assay, Miyata *et al.* [40] reported up to 65% reduction in DNA damage of rat liver exposed to 5 mg/kg AFB_1_ when treated with grapefruit juice intake for five days prior to mycotoxin exposure. To the best of our knowledge, the present study is the first one to employ the Comet assay to demonstrate that detoxification of aflatoxin-contaminated maize with NEW significantly diminishes AFB_1_-induced DNA damage in human lymphocytes.

In general, these results show the safety and efficacy of NEW to minimize aflatoxin-associated cytotoxicity and genotoxicity effects. For whole grain, it is likely that the detoxification treatment based on the use of NEW may be less effective than for ground maize, since toxins produced internally by the fungus are less likely to be exposed to NEW than toxins on the surface of the grain. However, this aspect remains under intense investigation.

## 3. Experimental Section

### 3.1. Safety Precautions

Household bleach containing 6% (*w*/*v*) sodium hypochlorite was applied to decontaminate surfaces of laboratory equipment, working areas and laboratory wastes. Wastes present in organic solvents were first evaporated to dryness. When solvent was dimethyl sulfoxide (DMSO), an equal volume of methylene chloride was added and then evaporated to dryness. Aflatoxin-contaminated materials (glassware) were immersed in the bleach solution overnight and then washed.

### 3.2. Chemicals and Reagents

Aflatoxins, bovine serum albumin, 3-(4,5-dimethylthiazol-2-yl)-2,5-diphenyltetrazolium bromide, 5,5′-dithiobis-(2-nitrobenzoic acid), dimethyl sulfoxide (≥99.5% purity), ethidium bromide, ethylenediaminetetraacetic acid, fluorescein diacetate, hydrochloric acid, hydrogen peroxide, isopropanol, low melting point agarose, perchloric acid, phosphate buffered saline, regular agarose type IIA, RPMI-1640, 5-sulfosalicylic acid, 2-thiobarbituric acid (≥98% purity), trizma base and triton X-100 were purchased from Sigma-Aldrich Co (St. Louis, MO, USA). l-glutamine (100X), non-essential amino acids (100X) and penicillin-streptomycin (10,000 U/mL) were obtained from Life Technologies (Grand Island, NY, USA). Acetonitrile (HPLC grade), ethanol 96%, methanol, methanol (HPLC grade), methylene chloride, sodium chloride and sodium hydroxide were obtained from J.T Baker, Mallinckrodt Baker (Ecatepec, Mexico). Nonidet P-40 was obtained from Fluka Chemical Corp, (Buchs, Switzerland). Protease inhibitors cocktail was obtained from Roche Diagnostics (Indianapolis, IN, USA). Oxoid Nutrient Broth No. 2 was purchased from Oxoid Limited (Basingstoke, UK). The S9 (metabolic enzymes) was obtained from Molecular Toxicology Inc. (Boone, NC, USA). All other chemicals used were analytical reagent grade.

### 3.3. Preparation of NEW

NEW was produced from electrolysis of a continuous supply of room temperature saturated NaCl solution diluted in tap water (~1% NaCl) using two patented generators from Esteripharma SA de CV (Atlacomulco, Mexico). NEW was used immediately after elaboration. Three physicochemical properties of NEW were verified and compared with those of DW, which was used as a control solution (Table 1). The pH and ORP values were measured with a combination pH/ORP/temperature meter (Hanna Instruments, Woonsocket, RI, USA; model HI-98121). The ACC was determined using a Chlorine Ultra High Range ISM meter (Hanna Instruments, Melrose, MA, USA; model HI96771C).

### 3.4. Maize Grain

Regular white maize of the commercial hybrid AS-900 provided by Aspros Comercial SA de CV (Cortazar, Mexico) was utilized. The grain was aflatoxin-free, as tested with the Association of Official Agricultural Chemists (AOAC) immunoaffinity column method described below. Moisture content was determined by drying replicate portions of 5 to 10 g each of whole grain at 103 °C for 72 h, with percentages calculated on a wet-weight basis.

### 3.5. Aflatoxin Production

Aflatoxins were produced according to the technique proposed by Méndez-Albores *et al.* [16]. The inoculated maize was incubated during 27 days to obtain an aflatoxin content of 360 ± 10 ng/g.

### 3.6. Aflatoxin Quantification

Aflatoxin content was determined according to the 991.31 AOAC method [41] using antibody-based immunoaffinity columns (IAC) for aflatoxin B_1_ and B_2_ (VICAM, Milford, MA, USA). Samples (50 g) were extracted by blending with 100 mL methanol-water (80:20, *v*/*v*) and 5 g of NaCl using a laboratory blender (Waring, New Hartford, CT, USA; Mod 51BL30). The mixture was filtered through a Whatman 1 filter paper and a 5 mL portion was diluted with 20 mL of DW. The diluted preparation was filtered through a micro-fiber filter, and 10 mL were passed through the IAC (Afla B, VICAM Science Technology, Watertown, MA, USA). Subsequently, the column was washed twice with 10 mL of DW and dried with sterile air flow. The toxins were then eluted with 1 mL of HPLC grade methanol and quantified in a fluorometer VICAM Series-4EX (VICAM Source Scientific. Irvine, CA, USA) after reaction with 1 mL of 0.002% aqueous bromine. The detection limit for aflatoxins via fluorescence measurement is approximately 0.5 ng/g.

### 3.7. Aflatoxin Identification

Aflatoxin identification was carried out by means of a Waters ACQUITY Ultra Performance Liquid Chromatography (UPLC) H-Class System equipped with a quaternary solvent manager (QSM) and an ACQUITY UPLC BEH C_18_ phase reverse column (2.1 × 100 mm, 1.7 µm). Standards, as well as samples collected from the IAC (1 µL) were injected and eluted with a single ternary mixture of 64:18:18 water/methanol/acetonitrile (all HPLC grade) at a flow rate of 400 µL/min. Aflatoxins were fluorometrically detected and identified using an UPLC-optimized fluorescence (FLR) detector (Waters, Milford, MA, USA). The excitation and emission wavelengths were 365 and 429 nm, respectively. Aflatoxins were identified by their Rt and compared with those for a pure aflatoxin standard solution under identical conditions. The estimated detection limits are 0.58 and 2.01 ng/kg for AFB_2_ and AFB_1_, respectively.

### 3.8. Detoxification of Aflatoxin-Contaminated Maize with NEW

The aflatoxin-contaminated maize containing 360 ± 10 ng/g was soaked in freshly prepared NEW (ACC 60 mg/L) for 15 min at room temperature. The ratio of liquid to solid was 3:1 and the batch size of the material treated was 1 kg. DW was used as a control solution under similar conditions. The soaked maize was filtered through a micro-fiber filter to remove excess water, and then dried at room temperature for 2 h in a sterile air flow chamber. The final moisture content of the treated maize was approximately 12%. Samples were tested for aflatoxins with the AOAC immunoaffinity column and UPLC methods as previously described.

### 3.9. Determination of Cytotoxicity

Aflatoxins cleaned up with IAC were transferred to amber vials and evaporated to dryness at 50 °C under stream of nitrogen and then redissolved in DMSO. Samples were filtered through Acrodisc 0.22 μm syringe filters (Gelman Sciences Inc., Ann Arbor, MI, USA) for sterilization. Under these particular conditions, the effects of aflatoxins or any antibody cross-reactive aflatoxin conversion products were evaluated for their cytotoxic and genotoxic potential.

#### 3.9.1. Cell Culture

The HCC epithelial cell line (HepG2) was obtained from American Type Culture Collection (ATCC, Manassas, VA, USA). HepG2 cells were routinely grown in monolayer culture in 10 mL of RPMI-1640 medium supplemented with 10% fetal bovine serum, 1% non-essential amino acids, 1% l-glutamine (200 mM), and 1% of a mixture penicillin (100 IU/mL)/streptomycin (100 μg/mL). Cells were maintained in 75 cm^3^ culture flasks under sterile conditions in an incubator at 5% CO_2_ and 37 °C.

#### 3.9.2. Cell Viability (MTT Assay)

Cell viability was carried out according Mosmann’s method [42]. After cell exposure with aflatoxins, the cell viability was measured by the MTT assay. This method determines the cells’ ability to convert tetrazolium salts to formazan dye by the succinate-tetrazolium reductase—a mitochondrial enzyme—which is active only in viable cells. The amount of dye produced is proportional to the number of live metabolically active cells. HepG2 cells (10^4^ cells/well) were seeded and maintained in 12-well plates (Corning Inc. Life Sciences., Tewksbury, MA, USA). Subsequently, confluent cell cultures (90% confluency) were exposed to aflatoxins (untreated and treated with NEW at 6 ng/mL) for a period of 4 h (this time was chosen to monitor acute cytotoxic effects on this particular cell line). Hydrogen peroxide at 30 mM was used as a positive control. After 2 h of incubation, the MTT solution (5 mg/mL) was added and plates were incubated to complete 4 h in humidified 5% CO_2_ at 37 °C. Afterwards, supernatant was removed and 1 mL of isopropanol solution containing 0.04 N HCl and 0.1% nonidet P-40 was added to each well before reading their optical density at 590 nm in a single cell Life Science UV-V is spectrophotometer Model DU530 (Beckman–Coulter Inc., Brea, CA, USA). The experiment was performed in triplicate.

#### 3.9.3. Lipid Peroxidation Assay

As a result of oxidative stress, cells produce hydrogen peroxide and aldehydes—due to peroxidation of lipids—which react with 2-thiobarbituric acid generating TBARS. Among these species, the generate MDA that can be quantified at 532 nm. The amount of the produced substance is proportional to the oxidative stress generated by lipid peroxidation. Lipid peroxidation was assayed according to the method of Buege and Aust [43] with minimal modifications. HepG2 cells were seeded on 6-well plates (10^4^ cells/well) until 90% confluence was reached. Confluent cell cultures were exposed to aflatoxins (untreated and treated with NEW at 6 ng/mL) for 4 h. Hydrogen peroxide at 30 mM was used as a positive control. After incubation, cell cultures were collected and rinsed with ice-cold phosphate buffered saline (0.1 M PBS, pH 7.2). Then, the cell suspension was sonicated in a volume of PBS containing a protease inhibitors cocktail and 0.1% triton X-100 using an ultrasonic cell disruptor model CPX130 (Cole Parmer, Vernon Hills, IL, USA) to obtain cell lysates. A volume of each sample was reserved to estimate the total protein concentration using the Bradford method [44], with bovine serum albumin as standard. The supernatant obtained was collected and mixed with ice-cold 2.5% perchloric acid to precipitate proteins. After centrifugation (Beckman-Coulter Inc., Brea, CA, USA; Microfuge 22R) at 2000× *g* for 15 min at 4 °C, 200 μL of sample was mixed with an equal volume of 0.67% (*w*/*v*) TBA, and the mixture was heated at 95 °C for 10 min in a water bath (Bellco Glass Inc., Vineland, NJ, USA). After cooling to room temperature, samples were submitted to spectrophotometric analysis at 532 nm. The concentration of TBARS was expressed in µmol per mg protein. Each experiment was performed in triplicate.

#### 3.9.4. GSH Assay

Reduced glutathione was also measured by spectrophotometric determination. The colorimetric method for analyzing total glutathione is based on the GSH recycling system by 5,5'-dithiobis-(2-nitrobenzoic acid) (DTNB) and glutathione reductase (GR). GSH and DTNB react to generate GSH, 2-nitro-5-thiobenzoic acid (TBN), and nicotinamide adenine dinucleotide phosphate (NADP^+^). Since TBN is a yellow colored product, glutathione concentration can be determined by measuring absorbance. HepG2 cells (10^4^ cells/well) were cultured on 6-well plates. Confluent cell cultures (90% confluency) were exposed to aflatoxins (untreated and treated with NEW at 6 ng/mL) for 4 h. Subsequently, cell cultures were scraped and collected in phosphate buffered saline (0.1 M PBS, pH 7.2). A sample of washed and sonicated cells was taken and a volume of 5% sulfosalicylic acid was added for protein removal. Samples were shaken, centrifuged (2000× *g*, 10 min, 4 °C) and the supernatant was reacted with DTNB [45]. The spectrophotometric analysis was done at 405 nm. GSH results were expressed in mmol per µg protein. Samples were tested in triplicate.

### 3.10. Determination of Genotoxicity

#### 3.10.1. Ames Test

The Kado micro–suspension assay—which is a simple and sensitive modification of the Ames test—was used [46]. For the micro-suspension procedure, histidine-requiring bacteria (*Salmonella typhimurium* tester strain TA-100) were grown overnight in Oxoid Nutrient Broth No. 2 to 1–2 × 10^9^ cells/mL. The cells were collected by centrifugation (4500× *g*, 10 min, 4 °C) and re-suspended in ice-cold phosphate buffered saline (0.15M PBS, pH 7.4). The S9 and S9 mix (metabolic enzymes and enzyme + cofactors, respectively) were prepared following the procedures of Maron and Ames [47]. The S9 (from Aroclor 1254 pre-treated male Sprague–Dawley rats) contained 40 mg protein/mL as determined using the Lowry *et al.* [48] method. The S9 concentration in the mix was 300 μg/mL. For the micro-suspension assay, 100 µL S9 mix, 100 µL concentrated bacteria in PBS (10^10^ cells/mL) and 10 µL aflatoxin standard (2.5, 5, 10 and 20 ng/plate) or 10 µL aflatoxins (untreated and treated with NEW at 5 ng/plate) were added to 12 × 75 mm sterile glass culture tubes kept on ice. The mixture was incubated in the dark at 37 °C with rapid shaking. After 90 min, the tubes were placed in an ice bath. Tubes were removed one by one, and 2 mL molten top agar containing 90 nmol histidine and biotin was added [35]. The combined solutions were vortex-mixed and poured on minimal glucose plates. Plates were incubated in the dark for 48 h at 37 °C. A colony counter (BioLogics Inc., Manassas, VA, USA) was used to count the number of revertant colonies. Strain markers and bacteria survival were routinely monitored. Interpretation of mutagenicity was done according to the two-fold rule [49], which states that a compound is significantly mutagenic if the test compound doubles the mean spontaneous mutation frequency. Samples were tested in triplicate for each independent experiment.

#### 3.10.2. Alkaline Comet Assay

The Comet assay was performed in alkaline conditions according to the procedure of Tice *et al.* [50] with minimal modifications. Blood samples were obtained by venipuncture with heparinized polyethylene syringes from three healthy male volunteers. Whole blood (20 µL) was cultured in Eppendorf tubes with 1 mL of RPMI-1640 media supplemented with 1% nonessential amino acids and 1% l-glutamine. The cells were treated with 4 µL aflatoxins (untreated and treated with NEW at 6 ng/mL) or 4 µL NEW alone. DMSO was used as a negative control and freshly prepared hydrogen peroxide at 30 mM as a positive control. After 3 h of incubation at 37 °C, an aliquot (100 µL) of each treatment was removed to evaluate cell viability as described subsequently. The remaining cells were centrifuged at 225× *g* for 2 min and the supernatant was discarded. The cell pellet was resuspended in 75 µL of low melting point agarose gel (0.5%) and dispensed on glass microscope slides (Waldemar Knittel Glass GmbH, Braunschweig, Germany) coated previously with 0.5% regular agarose. The low melting point agarose was allowed to set under a 24 × 50 mm cover slip (Waldemar Knittel Glass GmbH) by placing the slides at 4 °C for 10 min. The cover slips were then removed and another layer of low melting point agarose (75 µL) was added and allowed to solidify. The slides were processed simultaneously using a polyoxymethilene rack which was then left 18 h in ice-cold lysis buffer (100 mM disodium EDTA, 2.5 M·NaCl, 10 mM trizma base, pH > 10, containing 1% triton X-100 which was added freshly). The slides in the rack were then placed in ice-cold double DW for 30 min. Afterwards, the set of slides were placed in a high-throughput electrophoresis tank and covered with cold alkaline electrophoresis buffer (300 mM NaOH, 1 mM disodium EDTA, pH > 13) for 20 min. After alkali unwinding, the slides underwent electrophoresis at 27 V and 300 mA for 20 min, then rinsed twice with 0.4 M trizma base buffer (pH 7.5) and immediately fixed twice with ethanol 96%. In order to minimize DNA damage, all procedures were conducted under dim light. Finally, the slides were stained with 10 µL of ethidium bromide solution (1:10 in water) and 50 randomly selected cells per slide were analyzed with a fluorescent microscope Carl Zeiss Axio Scope.A1 (Gottingen, Germany), using the Comet Assay version IV software. The tail length parameter was used as an indicator of genotoxicity. Three independent experiments, each in triplicate, were performed.

##### Lymphocyte Viability

Cell viability in human lymphocytes was evaluated using simultaneous staining with fluorescein diacetate (FDA) and ethidium bromide (EtBr) according to the technique described by Jones and Senft [51]. The 100 µL aliquots of each treatment were transferred into new tubes and centrifuged at 225× *g* for 2 min; the supernatants were removed, and the cell pellets were kept on ice. Subsequently, the cell pellets were resuspended in 20 µL of FDA/EtBr solution, placed on slides and covered with cover slips. Blinded cell counts were performed with a fluorescence microscope Carl Zeiss Axio Scope A1 (Gottingen, Germany) using a 20× objective. Living cells were observed in green and dead cells in red; two-hundred cells were counted for each treatment.

### 3.11. Experimental Design and Statistical Analysis

The experiment was conducted as a completely randomized design. Data were assessed by analysis of variance (ANOVA) and means comparisons were performed according to the Tukey test using the Statistical Analysis System [52]. A significance value of *p* < 0.05 was used to distinguish significant differences between treatments.

## 4. Conclusions

Taken together, these data indicate that chemical treatment with NEW to detoxify aflatoxin-contaminated maize did not reduce the aflatoxin content in the treated grain (measured as loss of fluorescence) but significantly reduced their *in vitro* cytotoxicity and genotoxicity effects. However, more extensive *in vivo* assays need to be conducted to establish more confidently that the chemical detoxification process substantially reduces chronic aflatoxin–associated effects during exposure.

## Supplementary Materials

Supplementary materials can be accessed at: http://www.mdpi.com/2072-6651/7/10/4294/s1.
