# Supplementary material for: Detoxification of Aflatoxin-Contaminated Maize by Neutral Electrolyzed Oxidizing Water
Source: Toxins (Basel). 2015 Oct 23;7(10):4294–314. doi: 10.3390/toxins7104294 (PMC4626735; doi:10.3390/toxins7104294)
Supplement: Supplementary file 1 [file toxins-07-04294-s001.pdf]

Supplementary Materials

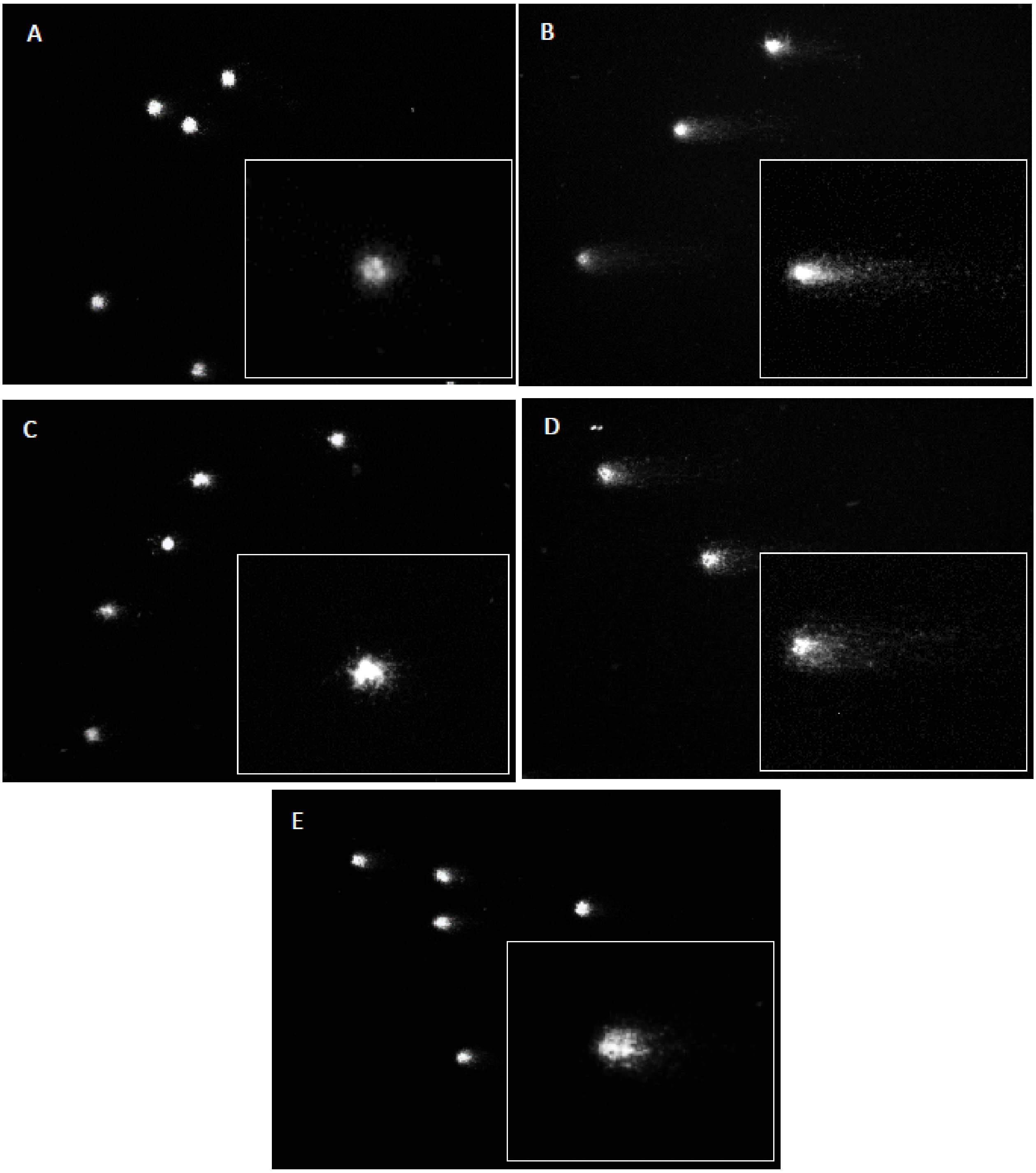

Figure S1. Representative comet images of human lymphocytes stained with ethidium bromide solution. (**A**) negative control (DMSO); (**B**) positive control (H_2O_2, 30 mM); (**C**) NEW (4 µL/mL); (**D**) untreated aflatoxins (AF at 6 ng/mL); (**E**), aflatoxins treated with NEW (AF + NEW at 6 ng/mL). Inset at higher magnification shows the differences in DNA migration pattern.
